# High DMBT1 concentrations in breast milk correlate with increased risk of infection in preterm and term neonates

**DOI:** 10.1186/1471-2431-12-157

**Published:** 2012-10-03

**Authors:** Sebastian Ronellenfitsch, Christel Weiß, David Frommhold, Lutz Koch, Jan Mollenhauer, Johannes Poeschl, Hanna Müller

**Affiliations:** 1 Division of Neonatology, Department of Pediatrics, University of Heidelberg, Im Neuenheimer Feld 430, 69120, Heidelberg, Germany; 2 Institute of Medical Statistics and Biomathematics, Medical Faculty Mannheim, University of Heidelberg, Ludolf-Krehl-Straße 13-17D, 68167, Mannheim, Germany; 3 Molecular Oncology and Lundbeckfonden Center of Excellence NanoCAN, Institute for Molecular Medicine, University of Southern Denmark, JB Winsloews Vej 25, 5000, Odense C, Denmark

**Keywords:** Breast milk, Deleted in Malignant Brain Tumors 1 (DMBT1), Neonatal infection

## Abstract

**Background:**

Human milk contains immune molecules involved in the protection of newborns against infections. We analyzed the concentration of Deleted in Malignant Brain Tumors 1 (DMBT1), a protein with functions in innate immunity, in breast milk.

**Methods:**

DMBT1 was detected in breast milk by Western blotting and its concentration was quantified by ELISA in 95 breast milk samples collected from mothers of preterm and term neonates during the first four weeks after delivery. Possible effects of maternal or neonatal parameters were analyzed by different statistical tests.

**Results:**

The mean DMBT1 concentration (± standard error of the mean) in the tested milk samples was 2.48 ± 0.26 μg/mL (range: 0.112 μg/mL to 17.984 μg/mL) and represented 0.0087% of the total protein content. The comparison between the newborns with infection and the newborns without infection revealed significantly higher DMBT1 concentrations in breast milk in the group with infection (6.72 ± 2.53 μg/mL versus 2.20 ± 0.35 μg/mL (*P* = 0.031)). Neither maternal nor neonatal parameters showed a correlation with the milk DMBT1 levels.

**Conclusions:**

DMBT1 is a component of breast milk after birth and is up-regulated in the breast milk from mothers with newborns suffering from neonatal infection. Thus, breast milk DMBT1 may be part of the innate immunity similar to secretory IgA.

## Background

The glycoprotein Deleted in Malignant Brain Tumors 1 (DMBT1), also known as glycoprotein 340 (gp-340) or as salivary agglutinin, is a member of the scavenger receptor cysteine-rich (SRCR) proteins with functions in innate immunity and epithelial differentiation [1,2]. Up-regulation of DMBT1 was observed in different tissues with inflammation [3-5]. The protein DMBT1 interacts with various defense factors such as, e.g., surfactant protein A and D and secretory IgA [1]. DMBT1 is able to directly bind to and aggregate various bacteria, which is sufficient to substantially suppress bacterial infection *in vitro*[6]. The broad bacterial-binding specificity is at least in part based on DMBT1 functioning as a pattern recognition molecule for poly-sulfated and poly-phosphorylated ligands [7].

In newborns, innate immunity is of particular importance, because the function of the adaptive immune system is not yet well established at these early time points of life. Especially premature infants benefit from the innate immunity because the delivery of IgG from the mother through the placenta to the fetus approximately begins in the second trimester, which is interrupted by the premature birth. On the other hand, neonatal infection and sepsis contribute largely to morbidity and lethality during early life [8-11].

The *DMBT1* gene has further been suggested as a tumor suppressor gene for different cancer types [12]. Several studies reported a reduced DMBT1 expression in breast cancer and a variable expression in normal breast tissue [13-15]. Strong expression upon inflammation was seen in some cases [15]. Possibly, *DMBT1* polymorphisms associated with changes in the number of SRCR domains and/or promoter activity contribute to these patterns [16,17]. However, in mammary gland tissues, DMBT1 is expressed in the epithelium of the mammary ducts and glands, which are also responsible for breast milk production. Breast milk is known to contain different proteins with functions in innate immunity such as lactoferrin and secretory IgA with beneficial functions in newborns [18-20]. We therefore hypothesized that breast milk may contain DMBT1 and that DMBT1 levels in breast milk could potentially correlate with infections in the neonates. To test this hypothesis we examined the DMBT1 concentrations in breast milk from mothers after delivery and tested whether the DMBT1 concentrations correlated with maternal and neonatal parameters.

## Methods

### Patients and samples

**Figure 1:**
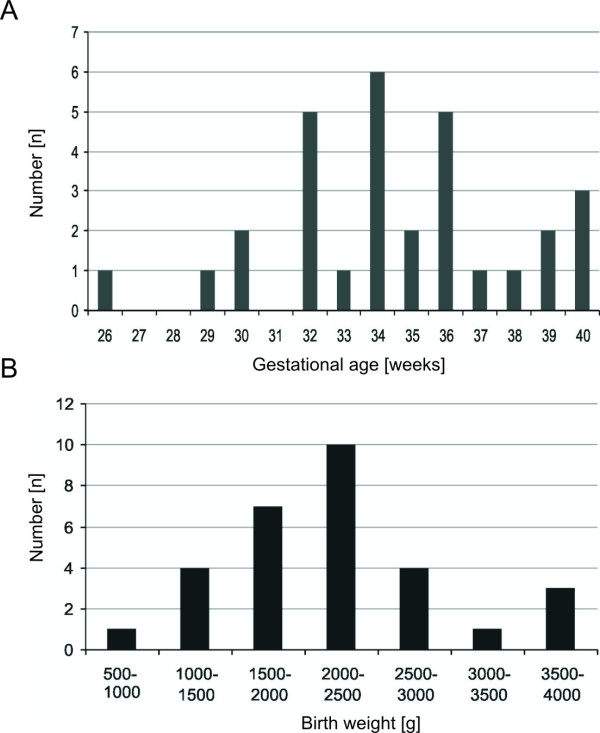
The distribution of the gestational age (A) and weight (B) at birth of the included infants.

The study was performed with approval of the responsible Ethics Committee of the University of Heidelberg, Germany, and in compliance with the Helsinki Declaration. The parents agreed by informed consent. Thirty mothers who delivered at the Perinatal Center of the University Hospital Center of Heidelberg were studied prospectively. The clinical data of these mothers are demonstrated in Table 1. Four mothers had twins, in these cases we included only the first born infant in the study. The included newborns comprised 14 females and 16 males. Gestational age was defined as time elapsed between the first day of the last menstrual period and the day of delivery. The included infants had an average gestational age of 34.5 ± 0.62 weeks (mean ± SEM; range: 26 – 40 weeks) (Figure 1A). The averaged weight at birth was 2225 ± 136.6 g (mean ± SEM; range: 590 – 3600 g) (Figure 1B). A sample of 1 mL fresh milk obtained by a breast milk pump was taken for determination of the DMBT1 levels once a week in the first four weeks, beginning on day 4 after delivery. All samples were left unpooled and stored immediately at -20°C until the analyses were performed. From 19 mothers all 4 samples were collected, while from 3 mothers only 3 samples, from 2 mothers two samples and from the remaining 6 mothers only one sample was obtained. This gave rise to a total of 95 samples, which were examined.

**Table 1:** Characteristics of the mothers

**Parameter**	
Maternal age (years; mean and range)	30.2 (21-39)
Number of pregnancies (mean and range)	2 (1-6)
Parity number (mean and range)	1.5 (1-6)
Mothers with abortion(s) (n)	10
Mothers with pre-eclampsia (n)	1
Mothers with HELLP syndrome (n)	3
Antiphospholipid antibody syndrome (n)	1
Maternal infection (n)	9
Antenatal steroids (yes/no)	13/17
Rupture of membranes (in hours prior delivery, mean and range)	2.9 (0-22)
Mode of delivery (vaginal delivery/cesarean section)	7/23

The DMBT1 concentrations of the breast milk measured for each mother were tested for correlation with clinical data and diseases of the newborns. The analysis included gestational age (weeks), birth weight (g), premature rupture of the membranes, increased C-reactive protein of the mother, maternal leukocytosis, prenatal group B *Streptococci* colonization of the mother, neonatal infection (C-reactive protein >10 mg/L, clinical signs of infection and consecutive therapy with antibiotics), respiratory distress syndrome, surfactant application, mechanical ventilation, therapy with continuous positive airway pressure (CPAP), and the presence/absence of persistent ductus arteriosus.

### Sodium dodecyl sulphate-polyacrylamide gel electrophoresis (SDS-PAGE) and western blot analysis

Separation of breast milk proteins was performed under non-reducing conditions on 8% polyacrylamide gels. The proteins were transferred onto nitrocellulose membranes (Whatman, GE Healthcare, Munich, Germany). The membranes were then incubated with the DMBT1-specific monoclonal antibody Hyb213-06 (Antibodyshop, Dianova, Hamburg, Germany) or the polyclonal anti-serum anti-DMBT1p84 [3]. After washing with Tris-buffered saline containing 0.1% Tween 20 (Gerbu, Gaiberg, Germany) (TBS-T) the membranes were incubated with the respective secondary antibodies (SC-2005, goat anti-mouse IgG, or SC-2004, goat anti-rabbit IgG, both conjugated with horseradish peroxidase, Santa Cruz Biotechnology, Munich, Germany).

### Analysis of the DMBT1 concentration in breast milk by enzyme-linked immunosorbent assay (ELISA)

Microtiter plates (Microcolon, Greiner Bio-One GmbH, Essen, Germany) were coated with breast milk over night at 4°C. Purified human recombinant DMBT1 (hrDMBT1) [21] was used as concentration standard. The plates were washed with TBS-T and incubated with the DMBT1-specific antibody Hyb213-06 (Antibodyshop, Dianova, Hamburg, Germany) followed by an incubation step with the secondary antibody AP 300P (sheep anti-mouse IgG, horseradish peroxidase-conjugated, Chemicon International, Temecula, California, USA). The bound enzyme was determined by administrating TMB-substrate solution [125 μg/mL 3,3^′^,5,5^′^-tetramethyl-benzidine; 125 μg/mL in 0.1 M citrate buffer pH 4.5 with 0.05% (v/v) H_2_O_2_. After 20 minutes the reaction was stopped by 2 M HCl and the intensity of the dye reaction was analyzed at 450 nm in an ELISA reader (Multiscan Ascent, ELISA Reader, Thermo Fisher Scientific Inc., Rockford, Illinois, USA).

### Determination of the total protein content

The total protein content of the breast milk samples were analyzed with the Lowry protein assay as described by Polberger and Lönnerdal [22].

### Statistics

The statistical analysis was performed with the SAS software package, release 9.2 (SAS Inc., Cary, NC, USA). We calculated means and standard errors of the mean (SEM) for quantitative parameters. To analyze potentially time-dependent changes of the DMBT1 concentrations und to search for parameters influencing the DMBT1 concentrations we used a variance analysis for repeated measurements using the SAS procedure PROC MIXED as this procedure is very efficient in the case of missing values. For analysis of the time-dependent changes of the total protein content a linear regression analysis was performed (where the number of weeks was regarded as a quantitative variable). To test the correlation between two quantitative parameters, we calculated the Pearson’s correlation coefficient. A logistic regression was used to determine the influence of one or several parameters on a binary outcome (for examples the outcome “infection” with the values “yes” and “no”). To compare two samples sets we performed the Mann–Whitney *U* test (quantitative, non-normally distributed data). Test results with *P*-values less than 0.05 were regarded as statistically significant.

## Results

### Detection of DMBT1 in breast milk

**Figure 2:**
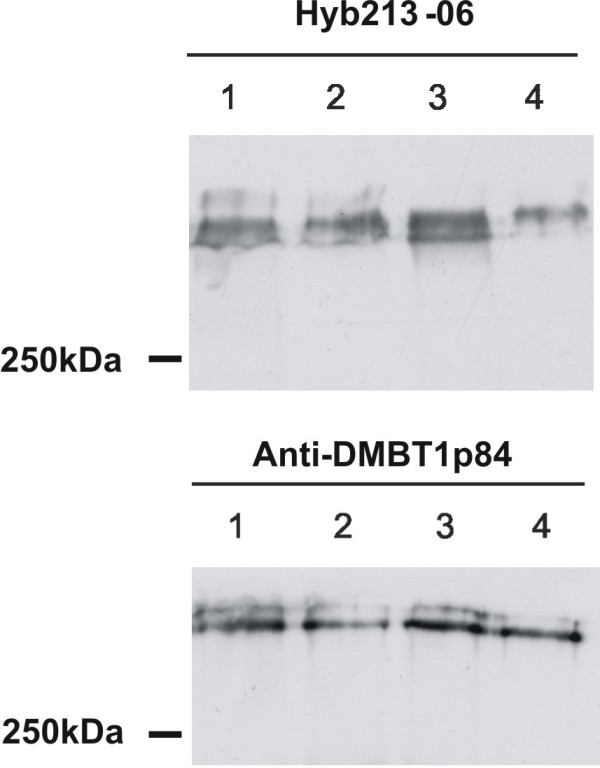
**Western blotting of breast milk from one exemplary individual included in the study.** Different antibodies against DMBT1 (Hyb213-06 or Anti-DMBT1p84) were used. The samples of week 1, 2, 3 and 4 after delivery (indicated with the numbers 1-4) were tested.

To test whether breast milk contains DMBT1, we performed Western blotting using different antibodies against DMBT1. DMBT1-specific bands with the expected size of approximately 340 kDa were readily detected by both antibodies (Figure 2). The observed double bands could be due to different glycosylation and/or different alleles of the *DMBT1* gene [16].

### Determination of the DMBT1 concentration in breast milk by enzyme-linked immunosorbent assay (ELISA)

**Figure 3:**
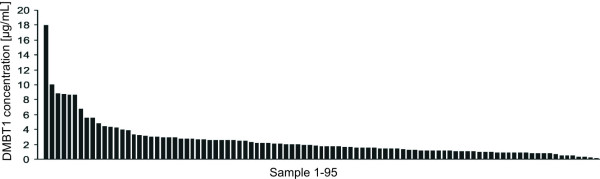
**DMBT1 concentrations of all 95 breast milk samples.** The samples were analyzed by ELISA using the monoclonal antibody Hyb213-06.

We used the enzyme-linked immunosorbent assay to quantify the DMBT1-content in the entire panel of breast milk samples. Figure 3 displays the DMBT1 concentrations, which were determined using recombinantly expressed DMBT1 of known concentration as the standard. The mean DMBT1 concentration in the breast milk samples was 2.48 ± 0.26 μg/mL (mean ± SEM) with a lowest DMBT1 concentration of 0.112 μg/mL and a highest of 17.984 μg/mL.

### DMBT1 concentrations in dependence of age (weeks after birth)

**Figure 4:**
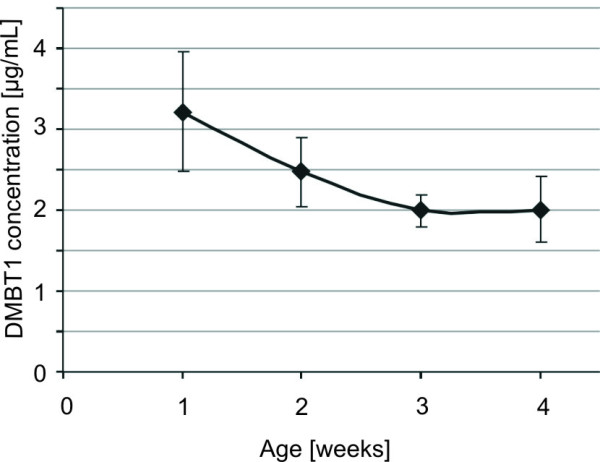
**DMBT1 concentrations (mean ± SEM) within the breast milk in the first four weeks of life.** Data included all 95 samples.

One sample per week 1-4 after delivery was analyzed, but there was no significant association between age (weeks after delivery) and DMBT1 concentration in the breast milk (*P* = 0.456; variance analysis for repeated measurements). The mean DMBT1 concentrations in the week 1-4 after birth are displayed in Table 2 and Figure 4. Numerically, the DMBT1 concentrations decreased after week 1 and 2 and reached a baseline in week 3 and 4.

**Table 2:** DMBT1 concentrations of breast milk in dependence of the time after delivery

**Week after birth**	**Week 1**	**Week 2**	**Week 3**	**Week 4**
mean DMBT1-conc. (μg/mL)	3.21	2.47	1.99	2.01
SEM	0.74	0.43	0.19	0.4
N	28	24	23	20

Conc. concentration.

However, because the time-dependent decrease was not statistically significant, we used the mean values of the samples obtained from each individual mother for testing the association of the DMBT1 concentration with different parameters.

### DMBT1 content in milk relative to the total protein content

**Figure 5:**
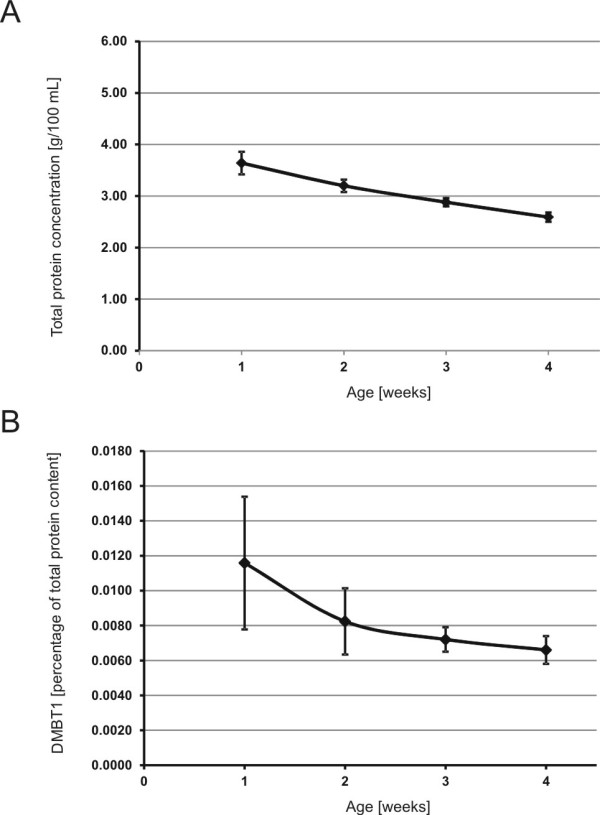
**DMBT1 and total protein content.** The total protein concentration (mean ± SEM) decreased in the first four weeks after birth (**A**). **B** illustrated the percentage of DMBT1 from the total protein content (mean ± SEM) in the first four weeks of life.

The mean total protein content of all included samples decreased linearly in an age-dependent manner (Figure 5A). Therefore, we used a linear regression analysis and found that total protein content decreased by about 0.366 g/100 mL on average per week (*P* < 0.0001).

The mean percentage of DMBT1 from the total protein content was 0.0087% (range: 0.0003 to 0.105%) and no significant changes during the first four weeks of age were observed (mean percentage; Figure 5B): week 1: 0.0116%; week 2: 0.0082%; week 3: 0.0072%; week 4: 0.0066% (*P* = 0.5244).

### DMBT1 concentration and gestational age at birth

The correlation analysis showed no significant association between the gestational age at birth and the DMBT1 concentration of breast milk (*P* = 0.479). The same applied, when using the mean value of the analyzed samples of each infant in week 1-4 (*P* = 0.520, r = -0.122, Pearson’s correlation).

### DMBT1 concentration and birth weight

The statistical tests did not identify a significant association between birth weight and DMBT1 concentration of the breast milk (*P* = 0.438; regression analysis and *P* = 0.223, r = -0.229; Pearson’s correlation with mean values of all samples of each infant).

### DMBT1 concentration and neonatal infection

**Figure 6:**
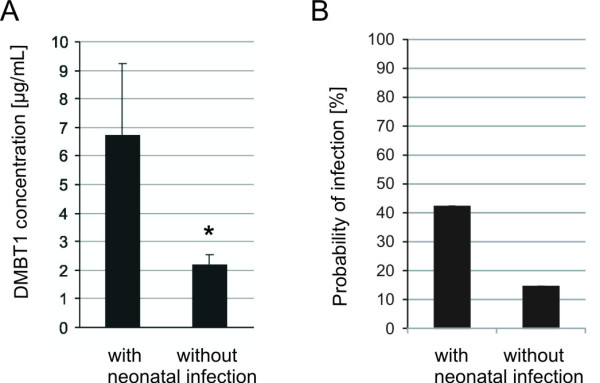
**DMBT1 concentration in breast milk and neonatal infections.** The DMBT1 concentration (mean ± SEM) in the breast milk from mothers of infants with neonatal infections versus infants without neonatal infection are demonstrated in **A**. The breast milk from mothers of neonates with infections showed significantly higher DMBT1 concentrations compared to the breast milk from mothers of neonates without infections (* *P* <0.05). **B** illustrates the calculated probability for an infection using the DMBT1 concentration-based mathematical equation for infants with versus without confirmed neonatal infection.

DMBT1 plays an important role in innate immunity [5,6,23]. Therefore, we tested the association between DMBT1 concentration in breast milk and neonatal infection. Six of the 30 (20%) included infants with 13 samples of breast milk had a neonatal infection (C-reactive protein >10 mg/L, clinical signs of infection and consecutive therapy with antibiotics). All neonatal infections manifested within the first seven days of life. *Staphylococcus aureus*, *Staphylococcus haemolyticus* and group B *Streptotococci* were observed in the cultures of blood and of the smears from the ears or the umbilicus. The statistical analysis using logistic regression and the mean values of all samples of each infant revealed a significant correlation between the DMBT1 concentrations in breast milk and the occurrence of neonatal infections (*P* = 0.044). The comparison between the newborns with infection and the newborns without infection revealed significantly higher DMBT1 concentrations within the breast milk of the group with infection (6.72 ± 2.53 μg/mL versus 2.20 ± 0.35 μg/mL (mean ± SEM; *P* = 0.031, Mann–Whitney *U*-test; Figure 6A)). The lowest mean DMBT1 concentrations in breast milk from mothers, which had infants with neonatal infections, was 1.838 μg/mL and the highest concentration was 17.984 μg/mL.

Here we provide a mathematical equation derived from a logistic regression to assess the probability of the newborn to have an infection for a distinct DMBT1 concentration in the breast milk.

In this case the DMBT1 concentration in the breast milk would be considered as a predictive marker for a neonatal infection:

(1) $$P=\frac{\exp\left(-2.7898+0.3968\cdot \text{DMBT1 conc}\right)}{1+\exp\left(-2.7898+0.3968\cdot \text{DMBT1 conc}\right)}$$

where *P* is the probability for a neonatal infection of the newborn and DMBT1 conc is the mean value of all measured DMBT1 concentrations in breast milk of each mother in the first four weeks of life. Using this formula, a *P*-value between 0 (0%) and 1 (100%) is guaranteed. The positive regression parameter b = 0.3968 indicates that the probability for the infection increases with higher DMBT1-values.

To test the predictive character of this formula we used the DMBT1 concentration in the respective mother’s breast milk to calculate the probability of neonatal infection for the infants of our study with versus without confirmed infections (Figure 6B). For the infants with confirmed infections we calculated a probability of 42.2% (mean; range: 11.3 – 98.7%) and for the infants without infection of 14.5% (mean; range: 7.4 – 69.7%).

Because the lowest mean DMBT1 breast milk concentration associated with an infected infant is 1.838 μg/mL, we assume that above this value the sensitivity for predicting a neonatal infection is 100%, but this would be accompanied by many false-positive predictions.

### DMBT1 concentration and other clinical parameters

The association between the DMBT1 breast milk concentration and different maternal parameters was evaluated with a logistic regression and the results are summarized in Table 3. Maternal infection or bacterial colonization and premature rupture of the membranes had no influence on the DMBT1 concentration. In addition, also the other neonatal parameters that were tested were without any significant association with the mean DMBT1 concentration (Table 3).

**Table 3:** Correlation between DMBT1 concentration of the breast milk and different maternal and neonatal parameters

**Parameter**	***P*-value**	**Test**
Maternal C-reactive protein >5 mg/L	0.167	Logistic regression
Maternal C-reactive protein >12 mg/L	0.313	Logistic regression
Maternal leukocytosis	0.460	Logistic regression
Premature rupture of the membranes	0.671	Logistic regression
Maternal group B streptococcal colonization	0.778	Logistic regression
Respiratory distress syndrome	0.100	Logistic regression
Surfactant application	0.062	Logistic regression
Mechanical ventilation	0.062	Logistic regression
Therapy with continuous positive airway pressure (CPAP)	0.072	Logistic regression
Persistent ductus arteriosus	0.102	Logistic regression

## Discussion

Breast milk consists of proteins, carbohydrates – especially lactose -, fat (97% triglycerides), vitamins, minerals, and viable cells, for example, leukocytes and macrophages [18-20]. The protein fraction comprises multiple bioactive proteins with diverse functions such as lactoferrin, Mac-2–binding protein, secretory IgA (sIgA), but many of these are related to defense functions. Earlier studies pointed to variable DMBT1 expression in normal breast tissue and a strong expression was detected in some cases with inflammation [13-15]. According to these studies, DMBT1 is expressed in the epithelium of the mammary ducts and glands, i.e. to structures, which are also responsible for the production and secretion of breast milk. We therefore considered it possible that DMBT1 could be secreted to breast milk after delivery. While Danielsson Niemi and co-workers could not detect DMBT1 in breast milk performing Western blotting with four different antibodies (mAb143, mAb303, Hyb213-01, Hyb213-06) against DMBT1 [24], our data point to the presence of DMBT1 in breast milk as confirmed by using two different methods (ELISA and Western blotting) and two different antibodies (Hyb213-06, anti-DMBT1p84). These findings strongly suggest that DMBT1 is one of the various breast milk components with functions in innate immunity, where it may function as a pattern-recognition molecule for pathogens and would co-localize with various of its known binding partners such as sIgA or lactoferrin.

The DMBT1 concentrations in our study ranged between 0.112 μg/mL and 17.984 μg/mL. The highest DMBT1 concentration was found in the first week after delivery, which then declines to an apparent steady-state level of approximately 2 μg/mL in week 3 and 4 after birth. This resembles the dynamics of other protective proteins such as lactoferrin, Mac-2 binding protein and sIgA in breast milk.

Lactoferrin, an interaction partner of DMBT1, has functions in the defense against bacterial and viral infections and was found in concentrations up to 3.3 mg/mL [19,25,26]. Montagne et al. found the highest lactoferrin concentrations directly after birth (5.8 mg/mL), decreasing in the first consecutive days and then again increasing after day 28 [25]. The Mac-2 binding protein (Mac-2 BP) is - like DMBT1 - a member of the SRCR superfamiliy. Mac-2 BP is expressed in tissues containing cavity-lining secretory epithelia (stomach, gut). It can function as an immune-stimulatory and anti-infective agent. D’Ostilio [27] detected Mac-2 BP in human breast milk and showed an increase until days 2-3 postpartum (13.4 μg/mL to 79.2 μg/mL) followed by a decrease of the Mac-2 BP concentration to <10 μg/mL on day 6 postpartum. They additionally performed one measurement 4 weeks after delivery and found concentrations of 5.3 ± 4.8 μg/mL. The mothers and infants in this study were healthy and had no infections. Likewise, the highest levels of secretory IgA (sIgA) were found directly after birth (19.0 mg/mL) and a decrease was reported throughout the lactation period (mature milk: 1.1 mg/mL) [28]. Similarly, cytokine levels significantly decrease in mature milk in comparison to the first milk samples postpartum, but in patients with preeclampsia the high proinflammatory cytokine levels were persistent throughout lactation [29]. Thus, the DMBT1 concentrations in our study follow a similar course, but were lower than the detected concentrations of lactoferrin, Mac-2 BP, and sIgA. Assuming an average amount of breast milk of 120 mL per day in the first days after delivery for term neonates, these infants receive 385 μg/day DMBT1 (3.21 μg/mL x 120 mL/day). In extreme premature infants, the amount of breast milk in the first days of life was about 12 x 1 mL to 12 x 3 mL per day (depending on the gestational age of the infants) corresponding to 39 μg and 116 μg DMBT1 per day.

Interestingly, our results revealed that breast milk from mothers with newborns suffering from neonatal infections showed higher DMBT1 concentrations in comparison to the breast milk from mothers with healthy newborns. In contrast, no correlation was found between the DMBT1 concentration in the breast milk and a maternal bacterial infection or a risk factor for chorioamnionitis (premature rupture of the membranes). It is, however, documented that sIgA levels in breast milk increase in response to the maternal environment [30,31]. In this case, antigen exposure in the gut or the respiratory tract activates B cell trafficking to the mammary glands, which then results in IgA secretion and translocation of sIgA to the breast milk [30-32]. While the precise mechanisms behind the up-regulation of DMBT1 levels in breast milk remain to be determined, it resembles the observations made for the Mac-2 BP. Fornarini et al. showed a significant association between the Mac-2 BP concentration in breast milk and an acute respiratory infection of the newborn in the first 12 months of life. The infants were breast feeded for 4-5 months. Newborns with high Mac-2 BP levels in the breast milk of their mothers had a lower rate of respiratory infections indicating a protective effect [33]. Because we analyzed only neonatal infections in our study group and the breast feeding time considered in our study is substantially shorter, it is not possible with the present data sets to compare the effects of DMBT1 on the infections rate as was performed in the earlier study of Mac2-BP.

Because of the blood volume using for blood culture (0.5-1 mL) only one third of the infants with neonatal infections showed positive blood cultures. To compensate for possibly low sensitivity we used additional criteria for the diagnosis infection (C-reactive protein >10 mg/L, clinical signs of infection as fever, and/or detection of bacteria in smears e. g. omphalitis), but we cannot rule out that a very strong inflammation was interpreted as neonatal infection.

According to the present data, the use of the DMBT1 concentration in the breast milk as a marker for neonatal infection is limited. The DMBT1 concentration in the breast milk of newborns with neonatal infection has a range between 1.838 and 17.984 μg/mL. This large range and the crossover of this range with the range of the DMBT1 concentration of healthy neonates would result in a high rate of false positive predictions. One reason could be basal differences of the DMBT1 expression levels in the breast tissues of individual women [13-15], which may depend on variable promotor activity [17]. Thus, the DMBT1 concentration of the breast milk can potentially contribute to diagnose a neonatal infection, but it is not able to replace established methods such as measurement of C-reactive protein levels to determine a neonatal infection.

## Conclusions

The results of our study demonstrate that DMBT1, known as a protein with functions in innate immunity, is detectable in breast milk after delivery. Higher DMBT1 concentrations are found in the breast milk from mothers of newborns suffering from neonatal infection in comparison to those of healthy newborns suggesting that breast milk DMBT1 may be part of mechanisms to protect the newborns from infections. Although DMBT1 has been shown to directly bind to and aggregate bacteria and that this is sufficient to substantially suppress infection *in vitro*, its roles in innate immunity in breast milk remains to be determined in detail because part of its functions may rely on or be influenced by specific interaction partners in milk.

## Competing interests

JM holds two patents in the area of DMBT1. The other authors declare that they have no competing interests.

## Authors’ contributions

SR collected the samples for the study and performed the ELISA experiments. CW carried out the statistical analysis. DF participated in the preparation of the manuscript. LK and JP enabled and supported the analysis of breast milk samples. JM took part of writing the manuscript. HM designed the study, analyzed the data, wrote and drafted the manuscript. All authors read and approved the final manuscript.

## Pre-publication history

The pre-publication history for this paper can be accessed here:

http://www.biomedcentral.com/1471-2431/12/157/prepub
